# Application of Free and Encapsulated DNA Tracers in Surface Water Studies in Lithuanian Climatic Conditions

**DOI:** 10.3390/biom15060889

**Published:** 2025-06-18

**Authors:** Dominyka Švedaitė, Anastasija Kriučkova, Augustas Morkvėnas, Vitalijus Karabanovas, Gintautas Stankūnavičius, Vigilija Klima, Jaunius Urbonavičius, Rūta Ivanec-Goranina

**Affiliations:** 1 Department of Chemistry and Bioengineering, Vilnius Gediminas Technical University, LT-10223 Vilnius, Lithuania; 2 Institute of Geosciences, Vilnius University, LT-01513 Vilnius, Lithuania

**Keywords:** free DNA, encapsulated DNA, tracers, RT-PCR, alginate/chitosane capsules, surface water studies

## Abstract

The applicability of free and encapsulated DNA as tracers in surface water studies in Lithuanian climatic conditions was evaluated. Tracer DNA synthesis and analysis were performed using real-time polymerase chain reaction (RT-PCR). Alginate and chitosan were used to obtain the microcapsules with DNA, and their sizes were determined using an atomic force microscopy. The Murlė stream in the city of Vilnius was chosen for field experiments using the prepared tracers. It was found that both types of tracers may be applied to surface water studies, but the relative concentration recovery of encapsulated DNA tracers is 3–6 times higher than that of free DNA tracers. It was concluded that the alginate/chitosan capsules protect DNA from the sandy layer in Murlė stream, direct UV exposure and other environmental factors that could degrade DNA. To our knowledge, this is the first report about free and encapsulated DNA tracer application in surface water studies in Lithuania.

## 1. Introduction

Environmental water pollution is one of the biggest challenges in today’s world. It has detrimental effects to society, economy and human health. Tracing the migration paths of pollutants in water, soil and atmosphere is one of the important factors to solve the environmental problems. In this sense, traceability is defined as the use of labeled materials to trace a specific material in nature, its movement paths, and distribution [1,2,3].

Synthetic DNA tracers are increasingly used in hydrological studies to understand hydrological systems, track pollution sources, water flows, and interactions. These tracers have unlimited number of unique variants, which are obtained by randomly combining DNA nucleobases [4,5]. The DNA sequences used are synthetically generated sequences that do not occur naturally. Their use reduces the possibility of detecting naturally occurring DNA that could interfere with the analysis [6]. Synthetic DNA has become popular due to the almost unlimited number of unique nucleotide sequences available, high specificity, and sensitivity of detection [7]. Also, DNA amplification technologies, such as quantitative polymerase chain reaction, allow the detection even if a single DNA molecule [8]. Due to its uniqueness, these tracers ensure the ability to detect a wide variety of materials simultaneously at multiple points [1,4]. The disadvantages of free synthetic DNA tracers are related to their degradation when exposed to environmental factors. This is most often related to the length of the synthesized DNA. Although there is some evidence that the rate of DNA decay is different at internal and terminal positions [9], many researchers argue that the overall rate of DNA decay is the sum of the rates of damage to individual nucleotides, which are assumed to be approximately equal throughout the sequence [10,11,12,13]. Degradation is also affected by temperature, microbial activity, enzymes, and chemicals. A significant portion of free DNA tracers is lost in hydrological studies due to adsorption and biological uptake, which is also one of the disadvantages [14]. Encapsulation helps increase DNA stability and protect against degradation and environmental factors [8,15]. Encapsulated synthetic DNA exhibits reduced aggregation at various salt concentrations [16]. Most studies are focused on hydrophilic encapsulating materials, which, by protecting DNA from environmental factors, increase the hydrophilicity of the particle, thereby stabilizing the aggregation equilibrium [16,17,18,19]. In hydrological studies, encapsulated synthetic DNA tracer particles can be tracked over longer distances [20]. Also, the encapsulating material gives the particles a negative charge, which helps reduce adsorption, resulting in less loss and consequently smaller concentration fluctuations compared to free DNA [20].

DNA tracers are rapidly detected, even at low concentrations, using real-time polymerase chain reaction (RT-PCR) [20,21,22]. Real-time polymerase chain reaction is based on the increase in fluorescence during amplification and involves real-time monitoring of RT-PCR products, which facilitates and accelerates the research process by performing quantitative evaluation [23,24,25]. This method is characterized by high sensitivity, specificity, has a wide dynamic range, and can detect extremely low amounts of target sequences [26,27,28].

Unlike the previously published works that examine the application of free and encapsulated DNA tracers, the climate of Lithuania is of a mid-latitude transitional type, located between maritime and continental climates. This climate is characterized by four seasons: warm summers, cool spring and autumn, and cold winters [29]. To our knowledge, no studies using synthetic free DNA and encapsulated DNA have been performed in real natural conditions in Lithuania so far. Until now, groundwater flows in Lithuania have been extensively studied using tracers such as salts, fluorescent dyes or isotopes [30,31,32]. However, these tracers are often ecotoxic and have other disadvantages [33,34]. Therefore, the aim of this work was to evaluate the applicability of free synthetic DNA and encapsulated DNA as tracers in surface water studies in Lithuanian climatic conditions.

## 2. Materials and Methods

### 2.1. Free DNA Tracer and Analysis

The RT-PCR method (Rotor-Gene Q Real-time PCR Cycler, QiaGen, Hilden, Germany) is used to amplify the target single-stranded DNA. The synthetic sequence was selected according to the [35]. The target sequence consists of 72 nucleotides that is 5′–ATCACATTCGAGGTGTCCACTAGATCCCGCGTTTTGTACCATCAGTCATTGCGCAGGTCGGTTGGTGGTAAG–3′. For DNA amplification, sequence of direct primer is 3′–ATCACATTCGAGGTCTCCAC–5′, and that of reverse primer is 5′–CTTACCACCAACCGACCT–3′. Synthesized DNA sequences: target sequence, direct primer, and reverse primer were purchased from Thermo Fisher Scientific (Waltham, MA, USA). Working solutions of 0.01 mM were prepared by diluting the prepared stock 0.1 mM solution with nuclease-free water (Thermo Fisher Baltics, Vilnius, Lithuania). If not used immediately, they are stored at −20 °C.

Real-time RT-PCR conditions were selected based on the available sequence and according to [35]: Initial DNA denaturation 95 °C 7 min. 40 cycles. DNA denaturation 95 °C 10 s. Primer annealing 53 °C 15 s. Elongation 72 °C 2 min. Final DNA synthesis 72 °C 2 min. After selecting appropriate RT-PCR conditions, appropriate amounts of the constituent RT-PCR mixture reagents were mixed in Eppendorf tubes under sterile conditions: 12.5 μL PowerUp™ SYBR^®^ Green Master Mix (Thermo Fisher Baltics, Vilnius, Lithuania), 4.5 μL DNA template, 2 μL each of reverse and direct primers, and 4 μL of nuclease-free water, with totalreaction volume of 25 μL.

### 2.2. Determination of DNA Concentration

DNA concentration was calculated from a standard curve that is made using a DNA solution of known concentration. Samples of known concentration are selected from 24.4 μg/mL and 10× dilutions are made. A “cycle threshold” (Ct) was determined for each amplification curve, the number at which the curve crosses the fluorescence background threshold. This thresholdwas chosen high enough to avoid background noise in the fluorescence signal, but low enough to remain within the exponential part. Due to the exponential nature of RT-PCR, the logarithm of Ct and the known concentration of the standard curve at each point forms a straight line of the standard curve [36].

**Figure 1:**
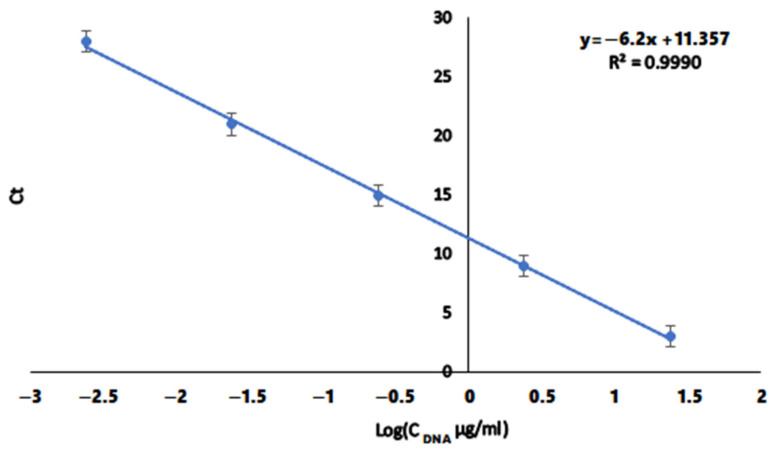
Standard curve to which all field experiments are recalculated. Ct is the cycle threshold, C_DNA_ is the standard DNA concentration (μg/mL).

The concentration of samples with unknown DNA concentration was calculated as follows: after RT-PCR, the Ct value is determined and after applying the standard curve formula (y = −6.2x + 11.357), the logarithmic value of the concentration was determined (Figure 1). All results were background-removed and concentration normalized as C/C_0_, where C is calculated DNA concentration after RT-PCR, C_0_ is the initial DNA concentration (120 μg/mL).

### 2.3. DNA Encapsulation and Control Experiments

**Figure 2:**
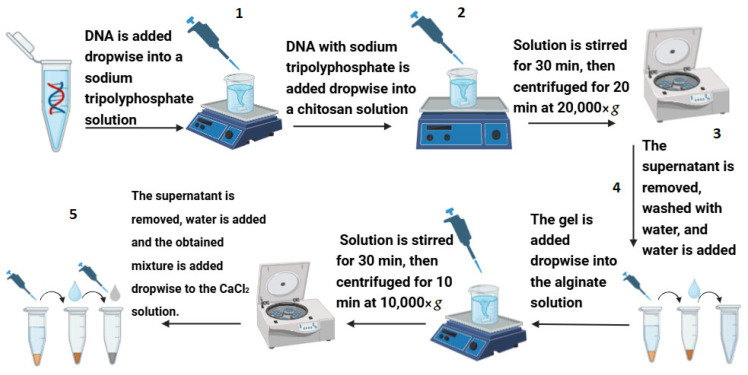
Synthetic DNA capsules production scheme according to [20].

After DNA amplification, DNA encapsulation was performed according to [20] (Figure 2). First, 150 μL of DNA was added to 30 mL of 0.125% sodium tripolyphosphate solution (Thermo Fisher Baltics, Vilnius, Lithuania), which was constantly stirred using a magnetic stirrer. The prepared DNA and sodium tripolyphosphate mixture is slowly added dropwise using a 100–200 μL pipette into a continuously stirred 0.5% chitosan solution (Sigma-Aldrich, Saint Louis, MO, USA). After all the DNA and sodium tripolyphosphate solution has been added, the resulting new mixture was stirred for another 30 min at room temperature. The resulting hydrogel was sedimented by centrifugation for 20 min at 20,000× *g* (Sigma centrifuge, Sigma Laborzentrifugen GmbH, Osterode am Harz, Germany), washed with deionized water three times. The hidrogel was diluted to 30 mL of deionized waeter, and 1 mL of it was slowly added dropwise by 100–200 μL into 30 mL of 1% sodium alginate solution (Sigma-Aldrich, Saint Louis, MO, USA) with stirring. The resulting new solution was stirred for another 30 min at room temperature. The formed capsules were centrifuged for 10 min 10,000× *g*. The supernatant was removed, the capsules are washed with deionized water and dropped into a 2% calcium chloride solution (Sigma-Aldrich, Saint Louis, MO, USA), which maintains the stability of the capsules and strengthens their protective alginate layer. The prepared capsules were used immediately in surface water tests or stored at +4 °C. The size of the produced capsules were determined using an atomic force microscope (the Innova Atomic Force Microscope, Bruker Corporation, Billerica, MA, USA).

To determine the encapsulation efficiency, capsules permeability measurements were performed. At the main encapsulation steps (marked with numbers in Figure 2), a 4.5 μL sample was taken and analyzed by RT-PCR. In each stage, DNA encapsulation occurs step by step, so free DNA in the samples taken should decrease. In the samples taken in the last stages, if no free DNA is found after RT-PCR analysis, it means that all DNA is encapsulated.

After encapsulating all the desired DNA and producing DNA capsules, they were stored in a 0.25 mM CaCl_2_ solution until further use. In order to ensure that the capsules remain stable and do not leak DNA into the solution over a longer period of time, a stability study of the capsules in a CaCl_2_ solution was performed. The background signal of the CaCl_2_ solution and the fluorescence of the CaCl_2_ solution with the capsules were monitored for eight consecutive days, which was then converted into DNA concentration.

In order to find out how the water of the Murlė stream affects the capsules, studies were conducted in the laboratory simulating field conditions—with constant intensive mixing of the Murlė water with the capsules.

Before starting work with encapsulated DNA in the field, optimization studies of DNA capsules disruption were carried out in the laboratory. A total of three different methods were used to achieve the best possible method of capsule disruption and DNA recovery: disruption using Chelex 100 for 24 h, disruption using Chelex 100 for 10 min, and disruption using acetic acid.

### 2.4. Field Experiments

The Murlė stream in Vilnius was selected for field experiments with DNA tracers. The stream has a groundwater source. In order to more accurately assess the impact of environmental and river conditions on field experiments, each time before the experiment, stream water was sampled for control and then the chemical composition of the water was assessed in the laboratory. In all cases, the chemical composition of the stream was very similar and did not change significantly. The content of nitrates (4–5 mg/L), nitrites (<0.003 mg/L) and ammonium (0.152–0.213 mg/L) in the water was minimal or at the detection limit, so their small amount in the water did not affect further studies. The amounts of total iron (248–253 μg/L) and manganese (53–57 μg/L) are small, judging by the Hygiene Norm, only minimally exceeding the norms set for drinking water (Fe^2+^ Fe^3+^ = 200 μg/L, Mn^2+^, Mn^3+^, Mn^4+^, Mn^7+^ = 50 μg/L). According to its general chemical composition, the stream water is clean and suitable for conducting experiments with free and encapsulated DNA tracers. Various measurements were also performed and recorded before each experiment. (Table 1).

**Figure 3:**
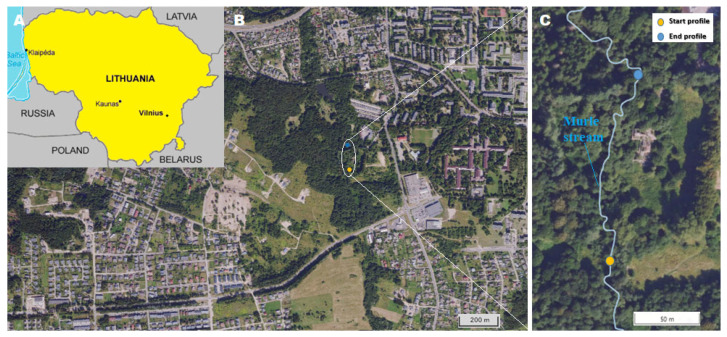
The site of the field experiment: (**A**)—map of Lithuania (source is www.google.com); (**B**)—part of the Naujoji Vilnia district of Vilnius city (source is https://uetk.biip.lt/zemelapis/ (accessed on 24 January 2025)); (**C**)—enlarged part of the Naujoji Vilnia district of Vilnius city, where the Murlė stream is visible (source is https://uetk.biip.lt/zemelapis/ (accessed on 24 January 2025)).

Before starting each experiment, the natural electrical conductivity of the stream was measured (a mobile conductometer Multi 3510 IDS, WtW Company, Wilheim, Germany). With both free DNA and encapsulated DNA tracers, a 10% NaCl solution was released during each experiment. NaCl solution is used as a control tracer and is released immediately before the DNA tracers. A 200 m stretch of the stream was selected from the tracer inlet to the sample collection site (Figure 3), and it was 0.8–1.6 m wide and 0.1–0.2 m deep. After performing a control experiment with only NaCl solution, it was determined that both NaCl and DNA tracers flow from the inlet to the collection point in approximately 15–30 min, depending on meteorological conditions. Samples are collected at the collection point every 1 min into 30 mL dark glass bottles; when an increase in the specific electrical conductivity of the river is observed, samples are collected every 30 s.

The collected samples were transported to the laboratory and filtered with 1 μm filters (Frisenette ApS, Viby, Denmark). Such filters are chosen because they allow both free DNA and encapsulated DNA to pass through, while not allowing larger stream water impurities that may interfere with further analysis. However, it is also known that some DNA can be adsorbed on the filter, therefore, when performing control DNA concentration calculations, all DNA solutions were also filtered, thus eliminating the sorption factor as much as possible. The filtrate is collected in 1 mL tubes and analyzed using further. The same procedure was performed with the control water of the Murlė stream without DNA tracers.

### 2.5. Disruption of DNA Capsules and Tracer Analysis of Free DNA

DNA capsules were disrupted using Chelex 100 (Sigma-Aldrich, Saint Louis, MO, USA). DNA capsules were suspended in 1 mL of 2% Chelex 100 solution, samples heated at 95 °C for 5 min with constant shaking 500 rpm (Digital thermoblock with shaking function, Ditabis AG, Pforzheim, Germany). Chelex resin protects DNA from degradation during boiling due to chelating metal ions, which can act as catalysts for DNA degradation at high temperatures in solutions of low ionic strength [37].

**Figure 4:**
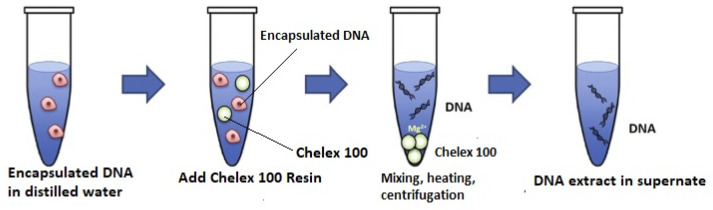
Schematic diagram of DNA release from microcapsules using method [38].

After 5 min, the parameters are changed to 300 rpm, 23 °C and left for 24 h similarly to [20], but with much longer shaking time to improve the DNA recovery. Afterwards, samples were centrifuged for 5 min at 10,000× *g*. The collected supernatant was analyzed by RT-PCR. A simplified scheme for capsules disruption is presented in Figure 4.

## 3. Results and Discussion

### 3.1. Determining the Size of DNA Capsules

**Figure 5:**
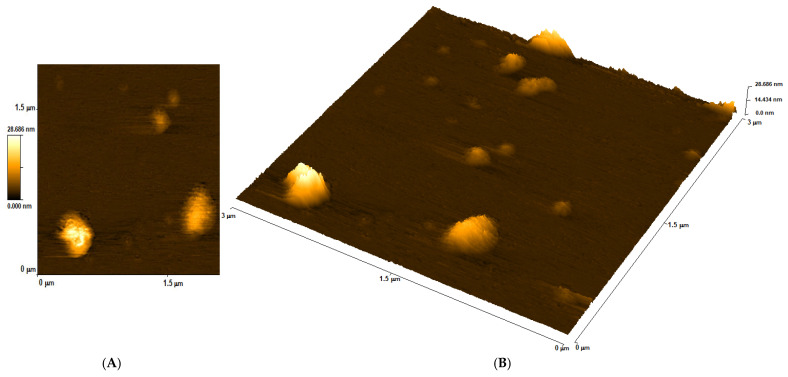
Atomic force micrograph of DNA-chitosan-alginate microparticles: 2D projection (**A**) and 3D projection (**B**).

Atomic force microscopy (Figure 5) showed that most of the microparticles were several hundred nanometers in size. The size of DNA-chitosan-alginate microparticles was obtained in the range from 100 nm to 500 nm. The morphology of the DNA capsules, resembling spheres in shape, was similar compared to [20].

### 3.2. Permeability and Stability of DNA Capsules

**Figure 6:**
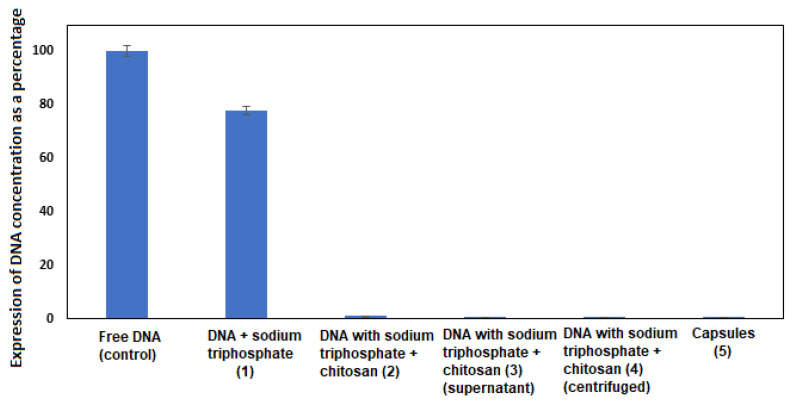
Permeability assay for DNA capsules. The numbers indicated next to each encapsulation step on the *X*-axis are related to Figure 2.

The results are presented in Figure 6. This study was performed to optimize the encapsulation conditions and minimize the percentage of DNA loss before conducting field experiments.

This study showed that in the first encapsulation stage, when DNA is dropped into a sodium triphosphate solution, compared to the control, 78% of DNA is detected (Figure 6). This result is normal, since DNA is not yet encapsulated, only distributed in the sodium triphosphate solution. In the second encapsulation stage, when a solution of sodium triphosphate with DNA is dropped into chitosan, DNA detection decreases to 3% before centrifugation. This change is influenced by the fact that a hydrogel is formed by the binding of the sodium triphosphate polyanion (phosphorus ion) to the chitosan cation (R-NH_3_^+^) under conditions of constant mixing. In all other stages (formed capsules, centrifuged gel and its supernatant), DNA is detected only up to 1%. This is a very good result, as it means that all the remaining DNA is encapsulated in chitosan and alginate capsules, which protect the DNA from entering the environment.

**Figure 7:**
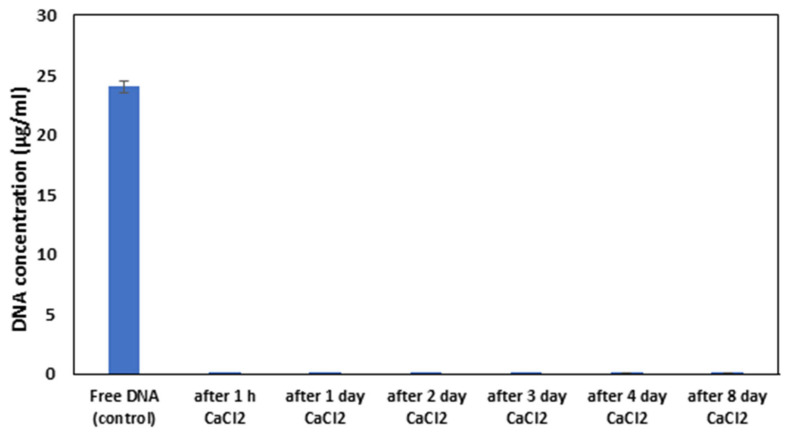
Stability of DNA capsules in 0.25 mM CaCl_2_ solution over time.

Another study of the stability of DNA capsules stored in CaCl_2_ solution also showed good results. In all cases, the values obtained did not exceed 0.0001 μg/mL. Compared to the control, this is 0.0004% (Figure 7). This study proves that the capsules remain stable and leak-proof for eight days when stored in a refrigerator in a 0.25 mM CaCl_2_ solution, so they are suitable for field experiments.

**Figure 8:**
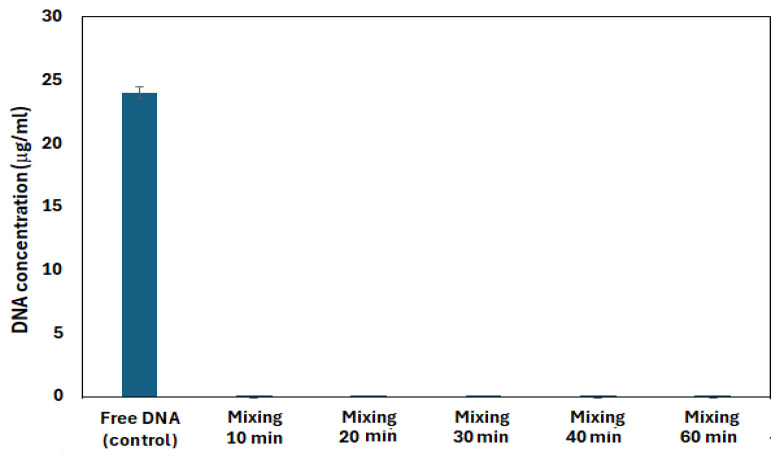
Stability of DNA capsules in the Murle stream water, simulating stream movement with stirring for 60 min.

Another study of the stability of DNA capsules in stream water, simulating the movement of the stream over time, also yielded good results. In this experiment, the detected DNA concentration varies from 0.0001 μg/mL to 0.004 μg/mL (Figure 8). Thus, very low (up to 0.02%) DNA loss from the capsules was observed when simulating flow activity. Despite this very small loss of DNA, it does not really interfere with the observation of DNA recovery and encapsulated DNA as a tracer is suitable for use in field experiments.

### 3.3. Disruption of DNA Capsules Under Laboratory Conditions

**Figure 9:**
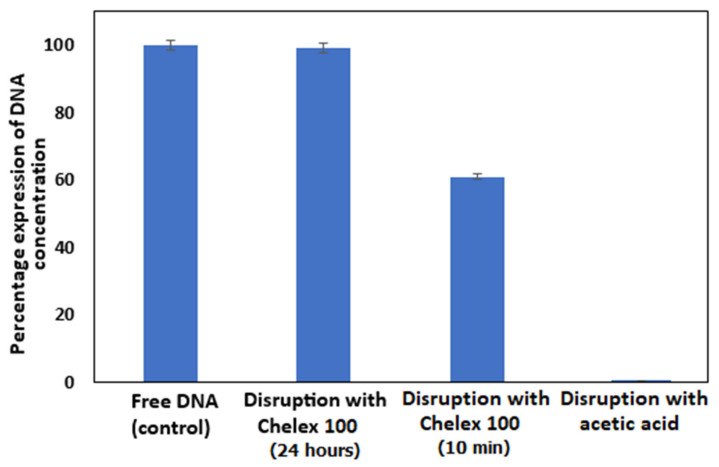
Optimization of DNA capsules disruption. Free DNA is used as a control. Disruption with Chelex 100 (10 min) according to source [20] and disruption with acetic acid according to source [39] were used. Concentration is expressed as a percentage compared to the control.

The results of the three different DNA capsules disruption methods are presented in Figure 9.

It can be seen from the study (Figure 9) that, compared to the control, the disruption with Chelex 100 according to the source [20] achieves only 61% DNA recovery (i.e., disruption for 10 min with constant shaking). Our adapted disruption method (i.e., disruption for 24 h with constant shaking) achieves a much higher DNA recovery rate of 99%. Disruption with glacial acetic acid was also attempted, based on the increase in chitosan solubility with the addition of acetic acid [39]. However, using this method, the disruption of the capsules failed. This happens most likely due to the alginate that forms the capsule shell, which, due to its structure and properties, is unable to be broken down and disrupted by acetic acid. After this study, further field experiments with encapsulated DNA tracers use disruption with Chelex 100, disrupting the capsules for 24 h with constant shaking.

### 3.4. Field Experiments with Free and Encapsulated DNA Tracers

**Figure 10:**
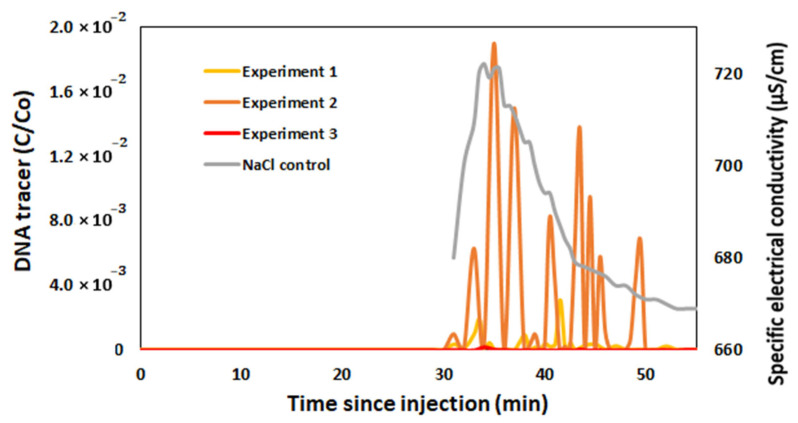
Relative concentrations of the free DNA tracers in surface water (NaCl tracer is used as a control).

A total of three successful field experiments with free DNA were performed where free DNA tracers could be recovered by RT-PCR method. The obtained experimental results are presented in Figure 10. As a sampling time control, NaCl solution was released during each experiment, helping to track when the DNA tracers released at the injection site reach the collection point. Although the results of all experiments differ, the frequency of DNA recovery and the difference in relative concentration are clearly different in experiment 3, which is very low. This could be due to the stream discharge, which is almost twice as high (23.1 L/s) as in experiment 1 (11.4 L/s) and experiment 2 (12.4 L/s). In experiment 1, the highest relative concentration of the DNR tracer (C/Co) was recovered to 0.003. Comparing all experiments, the highest relative concentration recovery was achieved in experiment 2, with 0.019, and the lowest was in experiment 3, with 0.0001. The first two experiments were conducted in the summer, and the third in the autumn, when temperatures were significantly lower. This may have affected the aggregation of the DNA tracer. Still, even such a small DNA recovery proves that free DNA tracers can be used in surface water studies.

**Figure 11:**
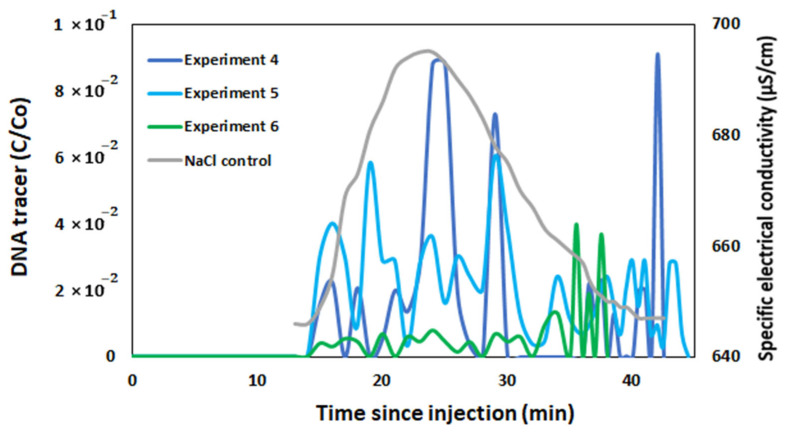
Relative concentrations of the encapsulated DNA tracers in surface water (NaCl tracer is used as a control).

Three successful field experiments with encapsulated DNA were also conducted. The results of all experiments are presented in Figure 11.

From the experiments performed with encapsulated DNA tracers, it can be seen that the highest relative DNA concentration was achieved in the Experiment 4 was 0.09 (stream discharge is 47.5 L/s). The relative concentrations of recovered DNA in Experiments 5 and 6 were lower, with the highest relative values ranging from 0.06 (flow rate is 32.6 L/s) to 0.04 (flow rate is 32.9 L/s). With encapsulated DNA tracers, there is a tendency that as the stream discharge decreases, the concentration of recovered DNA also decreases. This may be because the capsules are heavier, made of alginate and chitosan, so the slower stream discharge gives the capsules a greater chance to settle, adsorb on the stream bottom or edges in sandy media and not reach the sample collection point. Also, compared to the free DNA tracers studies, the stream flow rate was twice as high (Table 1) and this is reflected in the results (Figure 10 and Figure 11), as the encapsulated DNA tracers flowed twice as fast. All experiments with encapsulated DNA tracers were conducted in the spring, the temperature was similar and did not affect the studies.

**Figure 12:**
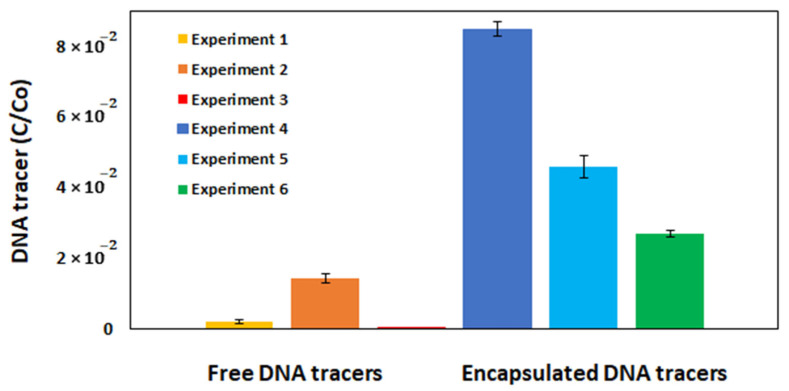
Comparison of average maximum relative concentrations of DNA tracers for each experiment determined during field experiments. Free DNA tracers—0.002 (Experiment 1), 0.014 (Experiment 2), 0.0001 (Experiment 3). Encapsulated DNA tracers—0.085 (Experiment 4), 0.046 (Experiment 5), 0.027 (Experiment 6).

Since free and encapsulated DNA tracers were not used in the same experiments, a direct comparison cannot be made, but a clear trend in favor of the encapsulated DNA tracer is seen (Figure 12). Although encapsulated DNA tracers were used in experiments where the stream flow rate was twice as high as for free DNA, the sampling time was also twice as early (Figure 10 and Figure 11). Therefore, it is expected that samples for both free and encapsulated DNA tracers were collected correctly, taking into account the stream flow. Another parameter that differed was temperature. In the case of encapsulated DNA tracers, lower water temperatures were more favorable and beneficial for capsule stability. Since higher temperatures could contribute to the swelling of chitosan-alginate capsules and thus contribute to the possibility of degradation [40]. Experiments with a free DNA (in our case single-stranded DNA) tracers were performed both at higher and lower temperatures. Better results were obtained at higher temperatures (Experiments 1 and 2). It is known from the literature that both high and low temperatures can contribute to single-stranded DNA aggregation [41]. However, more experiments with uniform field conditions should be performed in the future to better demonstrate the emerging trend.

In our case, although both encapsulated and free DNA tracers can be applied to surface water studies, the relative concentration recovery of encapsulated DNA tracers is 3–6 times higher than that of free DNA tracers (Figure 12). The same trend in DNA recovery when comparing free and encapsulated DNA has been observed in experiments by other researchers [20]. In experiments conducted by these researchers, it was observed that the recovery of encapsulated DNA is 2–3 times higher than that using free DNA tracers.

The studies conducted are just the beginning of further work. Before the routine use of DNA tracers in hydrological field studies can be started, many more systematic analyses need to be performed to see a better trend. Also, in the future, more studies should be repeated systematically at different times of the year in order to prove or disprove that the studies with DNA tracers in streams with a groundwater source are not affected by the seasons. In addition, although samples can be collected in the field and analyzed in laboratories, rapid detection of DNA tracers in the field would be very useful. It is expected that this will be possible in the future, as portable RT-PCR devices will become increasingly available over time. Thus, DNA tracer technology has a good prospect of facilitating hydrological studies in complex natural environments.

## 4. Conclusions

In this work, we obtained stable alginate/chitosan capsules with DNA tracers. We improved the method of disruption of such capsules for subsequent RT-PCR analysis by prolonging disruption with Chelex 100 chelating resin to 24 h with shaking. In this way, 99% efficiency of DNA recovery was achieved. We found that both free and encapsulated DNA tracers can be applied to surface water studies, but the recovery of the encapsulated DNA is 3–6 times higher than that of free DNA. We conclude that protecting DNA with alginate/chitosan capsules reduces the direct environmental and stream effects on its stability. To our knowledge, this is the first time when the field studies with synthetic DNA tracers have been conducted in Lithuania and demonstrate the feasibility of performing such experiments for investigating the surface waters.

## Figures and Tables

**Table 1:** Conditions of the surface water experiments.

Description, Units	Free DNA			Encapsulated DNA		
	Exp 1	Exp 2	Exp 3	Exp 4	Exp 5	Exp 6
DNA tracer volume, mL	1	1	1	1	1	1
DNA input, μg/mL	120	120	120	120	120	120
NaCl tracer, L	5	5	5	5	5	5
NaCl input, g/L	100	100	100	100	100	100
Date, month day	1 August	17 August	19 October	14 March	29 March	11 April
Temperature, °C	16.4	17.8	6.9	3.9	8.1	10.1
pH	6.61	6.72	6.57	6.71	6.9	6.63
Base conductivity, μS/cm	670	674	672	648	645	647
Stream flow rate, m/s	0.113	0.133	0.138	0.230	0.186	0.189
Stream discharge, L/s	11.4	12.4	23.1	47.5	32.6	32.9

## Data Availability

All data are contained within the article.
